# Impact of immunization against SpyCEP during invasive disease with two streptococcal species: *Streptococcus pyogenes* and *Streptococcus equi*

**DOI:** 10.1016/j.vaccine.2009.06.042

**Published:** 2009-08-06

**Authors:** Claire E. Turner, Prathiba Kurupati, Siouxsie Wiles, Robert J. Edwards, Shiranee Sriskandan

**Affiliations:** a Department of Infectious Diseases & Immunity, Hammersmith Hospital, Du Cane Road, London, W12 0NN, United Kingdom; b Department of Experimental Medicine & Toxicology, Imperial College London, Hammersmith Hospital, Du Cane Road, London, W12 0NN, United Kingdom

**Keywords:** *Streptococcus pyogenes*, SpyCEP, Vaccine

## Abstract

Currently there is no licensed vaccine against the human pathogen *Streptococcus pyogenes*. The highly conserved IL-8 cleaving *S. pyogenes* cell envelope proteinase SpyCEP is surface expressed and is a potential vaccine candidate. A recombinant N-terminal part of SpyCEP (CEP) was expressed and purified. AntiCEP antibodies were found to neutralize the IL-8 cleaving activity of SpyCEP. CEP-immunized mice had reduced bacterial dissemination from focal *S. pyogenes* intramuscular infection and intranasal infection. We also identified a functional SpyCEP-homolog protease SeCEP, expressed by the equine pathogen *Streptococcus equi*, which was able to cleave both human and equine IL-8. CEP-immunized mice also demonstrated reduced bacterial dissemination from *S. equi* intramuscular infection. Therefore immunization against SpyCEP may provide protection against other streptococci species with homologous proteases.

## 1 Introduction

Group A streptococcus (GAS) diseases are responsible for 500,000 deaths globally per year, mostly attributed to rheumatic fever and rheumatic heart disease, although cases of invasive GAS account for around 160,000 deaths [1]. There is currently no licensed vaccine for GAS. The surface M protein has been the main focus of GAS vaccine research and the most successful, but development has been hindered by the sheer number of different M-serotypes (>120 M-types) and cross reactivity with human proteins that may result in immune sequelae [2,3]. Current M protein-based vaccines use peptides derived from the conserved C-terminus [4,5] or multivalent peptides from the hypervariable N-terminus [6–8]. There have been many approaches to identify other suitable targets (reviewed in [9]). A whole genome bioinformatic and proteomic technique identified Spy0416 as a potential candidate, second only to M protein in conferring protection from death caused by an M23 strain [10]. Spy0416 is identical to the *Streptococcus pyogenes* cell envelope proteinase SpyCEP, and is responsible for cleaving and inactivating interleukin 8 (IL-8) [11]. SpyCEP is a highly conserved cell wall anchored subtilisin-like protease that is also released from the bacterial surface.

In this study, the potential for SpyCEP vaccination to protect against bacterial dissemination from both an intramuscular soft tissue infection and an upper respiratory tract infection caused by a clinically relevant M81 *S. pyogenes* strain, was evaluated in mice.

To determine whether SpyCEP represented a broad acting vaccine target we also identified functional SpyCEP homologs in the Lancefield group C strains *Streptococcus equi* and *Streptococcus zooepidemicus*. *S. equi* (*S. equi* subsp. *equi*) is a highly infectious equine pathogen that causes Strangles, a lymphadenitis of the head and neck resulting in abscessation and profuse nasal discharge, with occasional fatal dissemination to distant parts of the body (Bastard Strangles) [12]. Cross-protectivity of SpyCEP immunization against *S. equi* disease was also assessed. The work demonstrated that immunity to SpyCEP can prevent severe infection dissemination of two streptococcal species, and suggests potential for cell envelope proteases (CEPs) as vaccine candidates for all streptococcal species that express homologous proteases.

## 2 Methods

### 2.1 Bacterial strains

*S. pyogenes* strain H292 (M81) was isolated from a patient with rapidly progressive necrotizing fasciitis and bacteremia. Genome strains *S. equi* strain 4047 and *S. zooepidemicus* strain H70 were obtained from Andrew Waller (Animal Health Institute, Newmarket, UK). All streptococcal strains were cultured on Columbia horse blood agar or in Todd-Hewitt broth (THB) (Oxoid, Basingstoke, UK) at 37 °C, 5% CO_2_ without shaking. *Escherichia coli* were cultured in Luria-Bertani broth (Oxoid) with 50 μg/ml ampicillin (Sigma–Aldrich, Dorset, UK)

### 2.2 Recombinant CEP protein

A portion of the SpyCEP protein (CEP) encompassing amino acid residues 35–587 of the pre-pro SpyCEP sequence (Genbank No. DQ413032) was cloned and expressed using the pET100 cloning system (Invitrogen, Paisley, UK). This sequence includes two of the three active sites. Recombinant CEP protein was then nickel-column purified according to the manufacturer's instructions (Novagen, Nottingham, UK). Recombinant CEP was tested to ensure it had no serine protease activity and was used to raise rabbit polyclonal antiCEP serum [13].

### 2.3 Neutralizing activity of rabbit antiCEP serum

*S. pyogenes* H292 cell-free supernatant was co-incubated with 5 ng/ml human IL-8 (R&D, Abingdon, UK) and either 50% normal rabbit serum or antiCEP rabbit serum for 16 h at 37 °C. Uncleaved IL-8 was measured by human IL-8 duoset ELISA (R&D) (which does not detect cleaved IL-8) and IL-8 cleaving activity due to SpyCEP was calculated in comparison to the control (IL-8 in THB).

### 2.4 Flow cytometry

Approximately 1 × 10^8^ *S. pyogenes* H292 cells were harvested from an overnight culture and fixed for two hours at room temperature in 3% paraformaldehyde. After washing twice in PBS, cells were then incubated in 50 μl normal rabbit serum or rabbit antiCEP serum, diluted 1 in 10 in PBS, for 30 min at 37 °C. Cells were then washed twice in PBS-0.1% Tween20 before incubating with 50 μl of FITC labeled goat anti-rabbit IgG (Invitrogen) diluted 1 in 200 in PBS, on ice. Flow cytometry was performed on a FACSCalibur (Becton Dickinson, Oxfordshire, UK) counting 40,000 events. Data were analyzed using FlowJo software (Tree Star Inc.).

### 2.5 Immunization challenge

Eight-ten week old female BALB/c mice (Charles River, Margate, UK) weighing 20–24 g were immunized intramuscularly with 50 μg of recombinant CEP protein emulsified 1:1 in Freund's complete adjuvant (Sigma–Aldrich, Poole, UK). Two booster immunizations were given at days 14 and 21 in Freund's incomplete adjuvant (Sigma–Aldrich). A control group of mice were immunized with PBS and adjuvant. Tail bleeds were obtained on day 20. On day 28, mice were infected with *S. pyogenes* strain H292 or *S. equi* strain 4047. All animal procedures were conducted in accordance with UK Home Office guidance and approval. For intramuscular infection, mice received ∼5 × 10^6^ *S. pyogenes* and ∼5 × 10^7^ *S. equi* directly into thigh muscle. For upper respiratory tract infection, mice were anaesthetized with isoflurane and ∼3 × 10^8^CFU of *S. pyogenes* was allowed to be inhaled by placing 10 μl of bacterial suspension onto each nostril. After 24 h of infection, mice were euthanized and blood was taken by cardiac puncture. Spleen, liver and infected thigh muscle were excised from mice infected intramuscularly, individually weighed, and homogenized in PBS. For *S. equi* infection, only spleen and thigh was taken. Lung, nose and nasal-associated lymphoid tissue (NALT) in addition to spleen and liver, were taken from mice infected intranasally and homogenized in PBS. End points for infection were bacterial dissemination to blood, spleen or liver and IL-6 levels, measured by ELISA duoset (R&D), at 24 h. Infection dissemination was determined by culturing known quantities of blood, liver and spleen. Viable CFU were measured on horse blood agar.

### 2.6 Western blotting of culture supernatant and cell wall fractions

Streptococcal strains were grown to early (EL, A_600_0.2–0.3), mid (ML, A_600_0.5) and late (LL, A_600_0.7–0.9) log phases of growth. Cell wall preparations were made using an enzymatic method described by Biswas et al. [14]. Culture supernatant and cell wall extract proteins were separated by 10% Bis-Tris SDS-PAGE gels (Invitrogen) and immunoblotted using rabbit antiCEP serum. Blots were developed using goat anti-rabbit-HRP and the ECL system (GE Healthcare, Chalfont St Giles, UK).

### 2.7 IL-8 cleavage SDS PAGE and immunoblotting

Cell-free bacterial culture supernatant was incubated at 37 °C overnight with 100 μg/ml human IL-8 (R&D) or 100 μg/ml equine IL-8 (Perbio, Northumberland, UK). IL-8 was detected by 12% Bis-Tris SDS-PAGE (Invitrogen) stained with Colloidal Blue staining kit (Invitrogen) or immunoblotted with anti-human IL-8 HRP labeled antibody (R&D) and streptavidin (R&D).

### 2.8 Measurement of antibody responses to CEP

To measure murine antibody response to recombinant CEP an ELISA-based assay was employed. Plates were coated with 20 μg/ml CEP and bound mouse IgG was detected with HRP conjugated goat anti-mouse IgG.

### 2.9 Mass spectrometry

*S. pyogenes* and *S. equi* culture supernatants containing SpyCEP or SeCEP respectively, were concentrated by 100 kDa centrifugal filter devices (Amicon, Co Durham, UK). Chemokines human IL-8 (R&D) and equine IL-8 (Perbio) were suspended in acetonitrile:water:formic acid (20:80:0.1) and diluted to 40 ng/μl in 50 mM ammonium bicarbonate and co-incubated with 1 μl of concentrated supernatant at 37 °C for 18 h. Digested and undigested human IL-8 and equine IL-8 were chromatographed on a C4 picofrit column (0.075 × 10 mm) (New Objective, supplied by Presearch, Basingstoke, UK) and analyzed by electrospray MS using an LTQ Iontrap MS (ThermoFisher Scientific, Hemel Hempstead, UK); other conditions were as described previously [11].

### 2.10 Statistical analysis

Comparative statistics were performed using non-parametric analysis with GraphPad Prism version 4.03 for Windows, GraphPad Software, San Diego California USA. Chi-squared analysis was performed where groups of 10 or more were used.

## 3 Results

### 3.1 Polyclonal antiCEP antibodies recognize SpyCEP and are neutralizing

**Fig. 1:**
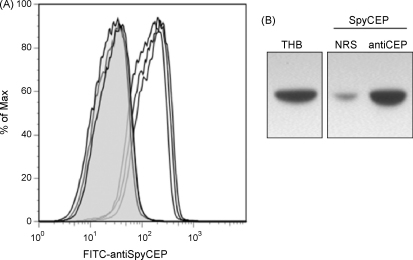
(A) Flow cytometric demonstration of rabbit antiCEP serum binding to surface SpyCEP using *S. pyogenes* strain H292. Cells stained with antiCEP (unshaded peaks) showed increased fluorescence compared to those stained with normal rabbit serum (shaded gray peaks) indicating binding of antiCEP antibodies to surface expressed SpyCEP. Experimental triplicates are shown. (B) Rabbit antiCEP serum neutralizes SpyCEP. Western blot for human IL-8. Culture supernatant from *S. pyogenes* was co-incubated with 5 ng human IL-8 in the presence of normal rabbit serum (NRS) or antiCEP rabbit serum (antiCEP). IL-8 incubated with THB alone (THB) was included as a control.

Rabbit antiCEP serum was able to recognize and bind to SpyCEP expressed on the surface of whole *S. pyogenes* H292 cells as determined by flow cytometry (Fig. 1A). This antiCEP serum was also able to neutralize the IL-8 cleaving activity of SpyCEP present in H292 culture supernatant (Fig. 1B). This suggested that recombinant CEP protein induced antibodies with the potential to provide protection from *S. pyogenes* infection.

### 3.2 CEP immunization reduced disease severity and dissemination in intramuscular *S. pyogenes* infection

**Fig. 2:**
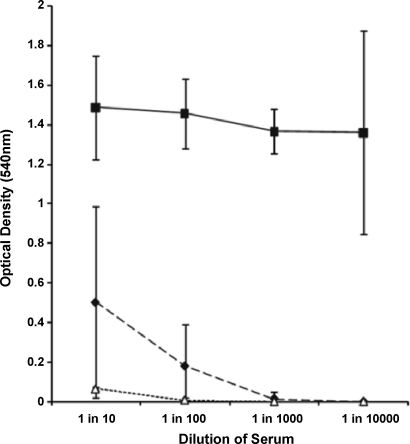
Immunization of mice with CEP induces antiCEP antibodies. Serum was taken on day 20 from mice immunized with recombinant CEP (solid line, squares) or PBS (dashed line, diamonds). Serum obtained from normal unimmunized mice was also included (dotted line, triangles). CEP specific IgG was measured using an ELISA-based assay in serum diluted in PBS. Data show the mean of sera from 6 mice (±standard deviation).

**Fig. 3:**
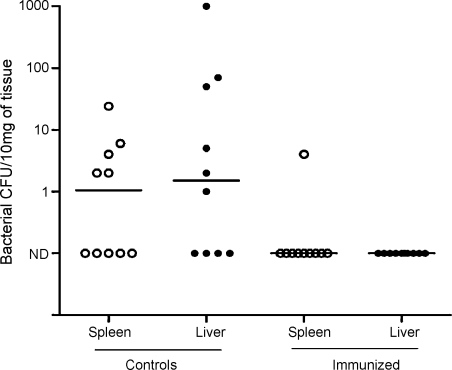
After challenge intramuscularly with 6 × 10^6^ CFU/mouse of *S. pyogenes*, 8/10 control mice demonstrated bacterial dissemination to liver (filled circles) or spleen (open circles), while only 1/10 CEP-immunized mice demonstrated dissemination (*χ*^2^ = 9.899; *p* < 0.001). Exact bacterial counts per 10 mg of organ from each mouse are shown; 3 mice in the control group showed dissemination to both liver and spleen. Median is represented by a horizontal bar. N.D: not detected.

Mice immunized with recombinant CEP had a consistently high serum IgG response, even at 1:10,000 dilution, compared with controls (Fig. 2). Following intramuscular challenge, infection dissemination to blood, liver or spleen occurred in 8/10 control mice but only 1/10 CEP-immunized mice (*χ*^2^ = 9.899; *p* < 0.001). Exact bacterial counts of spleen and liver are shown in Fig. 3. Only one mouse, from the control group, was bacteremic. Bacterial load in the thigh muscle was unaffected by CEP vaccination, possibly due to the short duration of the experiment (24 h) (Controls: median 3.8 × 10^6^ CFU/mg, range 1.0 × 10^6^ to 1.0 × 10^7^ CFU/mg. Immunized: median 6.8 × 10^6^ CFU/mg, range 1.0 × 10^6^ to 2.4 × 10^7^ CFU/mg).

Serum IL-6, a marker of disease severity, was lower in the CEP-immunized mice (median 91.3 pg/ml, range undetectable-1263.0 pg/ml) compared to the control mice (median 314.7 pg/ml, range 9.8–776.4 pg/ml; *p* = 0.0433). Taken together, spread of disease and disease severity were reduced by immunity to SpyCEP in this infection model.

### 3.3 CEP immunization reduced dissemination in intranasal *S. pyogenes* infection

**Fig. 4:**
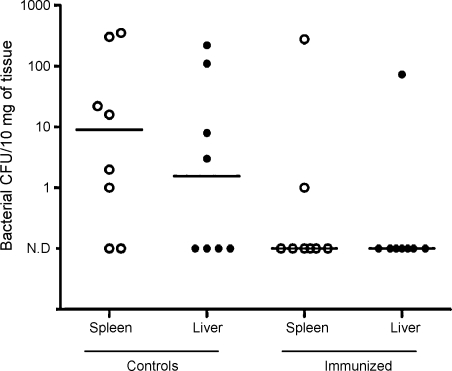
After challenge intranasally with 3.1 × 10^8^ CFU/mouse of *S. pyogenes* 6/8 control mice demonstrated bacterial dissemination to liver (filled circles) or spleen (open circles), while only 2/8 CEP-immunized mice demonstrated dissemination. Exact bacterial counts per 10 mg of organ from each mouse are shown; 4 mice in the control group and 1 mouse in the immunized group showed dissemination to both liver and spleen. *N* = 8 per group. Median is represented by a horizontal bar. N.D: not detected.

Following intranasal challenge, infection dissemination to blood, liver or spleen occurred in 6/8 control mice but only 2/8 CEP-immunized mice. Exact bacterial counts of spleen and liver are shown in Fig. 4. Two mice from each group were bacteremic. Although numbers in groups were small the data suggest that spread from the initial infection site was reduced by immunization with SpyCEP. Immunization with CEP did not, however, influence carriage of *S. pyogenes* in the nose and nasal-associated lymphoid tissue during the 24 h experiment (Table 1). Serum IL-6 was no different between CEP-immunized mice (median 311.3 pg/ml, range 57.0 pg/ml–1.4 ng/ml) and control mice (median 120.8 pg/ml, range undetectable-28.5 ng/ml).

### 3.4 Homologous proteases in *S. equi* and *S. zooepidemicus*

**Fig. 5:**
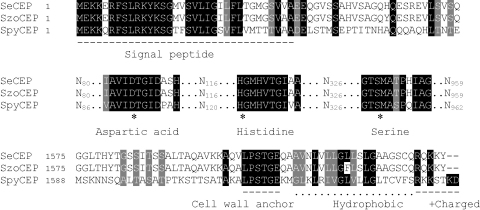
SeCEP and SzoCEP are homologous to SpyCEP. Identical residues are shaded black and similar residues are shaded gray. Putative signal peptides span residues 1–34. At the C-terminus all three proteins encode a sortase processing LPXTG motif which precedes a hydrophobic region and a positively charged tail for cell wall anchorage. The subtilase active residues aspartic acid, histidine and serine (indicated by (*)) and surrounding residues show 81% identity.

**Fig. 6:**
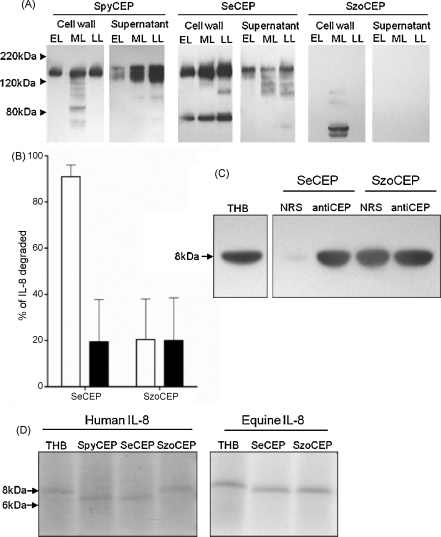
SeCEP and SzoCEP have IL-8 cleaving activity. (A) Western blot of culture supernatant or cell wall extract from *S. pyogenes*, *S. equi* and *S. zooepidemicus* to detect SpyCEP, SeCEP and SzoCEP respectively using antiCEP serum at different phases of growth (EL: early log, ML: mid log, LL: late log). Expression of SeCEP was similar to SpyCEP. (B) IL-8 cleaving activity of *S. equi* and *S. zooepidemicus* late log phase culture supernatant in the presence of normal rabbit serum (white bars) or rabbit antiCEP serum (black bars). Percentage of IL-8 cleaved was calculated in comparison to the IL-8 control (IL-8 incubated with THB alone). Data represent the mean of four separate experiments measured in duplicate (+standard deviation). (C) Western blot for human IL-8. Culture supernatants of *S. equi* or *S. zooepidemicus* were incubated with 5 ng of human IL-8 in the presence of normal rabbit serum (NRS) or rabbit antiCEP serum (antiCEP). IL-8 incubated in THB alone was used as a control. (D) Colloidal blue stained SDS PAGE for human IL-8 (left) or equine IL-8 (right) after incubation with culture supernatant from *S. pyogenes*, *S. equi* and *S. zooepidemicus*. IL-8 incubated in THB alone was used as a control.

We hypothesized that vaccination against SpyCEP could also protect against other streptococcal infections. A search of other streptococcal genomes revealed that the SpyCEP gene (*cepA*) has many homologs in other species but particularly in the Lancefield group C species *S. equi* and *S. zooepidemicus*. *S. equi* (Strain 4047, Sanger Institute) cell envelope proteinase (SeCEP) and *S. zooepidemicus* (Strain H70, Sanger Institute) cell envelope proteinase (SzoCEP) are 98% identical to each other by BlastP analysis and are 59% identical (74% similar) to SpyCEP. SpyCEP, SeCEP and SzoCEP all encode N-terminal signal peptides (predicted by SignalP 3.0 server) from residues 1–34 and a C-terminal LPSTG cell-wall anchoring motif for secretion of the enzyme to the cell surface and attachment to the cell wall (Fig. 5). The three catalytic domains typical of cell envelope proteinase containing the active residues aspartic acid, histidine and serine, have a high degree of sequence similarity. Using rabbit antiCEP serum, SeCEP was detected in the culture supernatant and cell wall extract of *S. equi* (Fig. 6A). SzoCEP could not be detected in culture supernatant or cell wall extract of *S. zooepidemicus* despite homology to both SeCEP and SpyCEP, suggesting poor expression in this strain. SeCEP was detected throughout exponential growth as a ∼160 kDa protein which is approximately the same size as the fully processed SpyCEP. A secondary band was also detected at ∼70 kDa, not present in the SpyCEP Western blot but it is unclear what this band corresponds to. Preimmune serum blots were blank (not shown).

Cleavage of human IL-8 by SeCEP and SzoCEP was measured by ELISA which demonstrated approximately 90% degradation of IL-8 by SeCEP that could be inhibited by antiCEP serum (Mann Whitney, *p* = 0.0286). *S. zooepidemicus* demonstrated limited cleavage of IL-8 that was unaffected by the presence of antiCEP serum (Fig. 6B). Specific IL-8 degradation by SeCEP was also detected by Western blot (Fig. 6C). Both ELISA measurement of IL-8 degradation and Western blotting for IL-8 demonstrated that SeCEP could degrade human IL-8 but SzoCEP did not. Degradation of human IL-8 appeared to be specific (Fig. 6D); SpyCEP- and SeCEP-cleaved IL-8 had approximately the same molecular weight. In contrast, cleavage of equine IL-8 by both SeCEP and SzoCEP was observed by SDS PAGE (Fig. 6D).

**Fig. 7:**
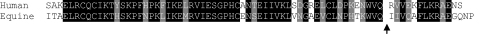
Human and equine IL-8 cleavage. Alignment and BOXSHADE of human and equine IL-8 where identical residues are shaded black and similar residues are shaded gray. The point of cleavage of both IL-8 molecules by SpyCEP and SeCEP is indicated by a vertical arrow.

The site of cleavage of human IL-8 and equine IL-8 by SeCEP was confirmed by mass spectrometry (Fig. 7). SeCEP cleaved human ^1–72^IL-8 (*M*_av_, 8387.00) at the same position as SpyCEP; between ^59^Q and ^60^R to form ^1–59^IL-8 (*M*_av_, 6829.0). Cleavage of equine ^1–74^IL-8 (*M*_av_, 8377.49) by SeCEP occurred in the analogous position between ^59^Q and ^60^I, to form ^1–59^IL-8 (*M*_av_, 6711.90). SpyCEP was also confirmed to cleave equine IL-8 in the same position.

### 3.5 Immunization with recombinant CEP prevents dissemination of *S. equi* infection

**Fig. 8:**
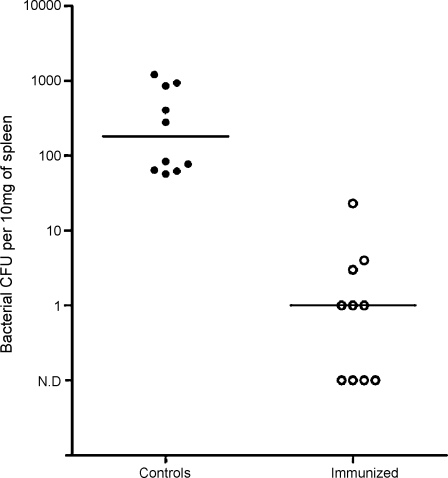
After challenge intramuscularly with 5 × 10^7^ CFU/mouse of *S. equi* strain 4047 10/10 control mice demonstrated bacterial dissemination to liver (filled circles) or spleen (open circles), while only 6/10 CEP-immunized mice demonstrated dissemination (*χ*^2^ = 5.0; *p* = 0.0127). Exact bacterial counts per 10 mg of organ from each mouse are shown. Control mice also had higher bacterial counts in the spleen than the immunized group (Mann Whitney, *p* < 0.0001). *N* = 10 per group. Median is represented by a horizontal bar. N.D: not detected.

Following intramuscular challenge with *S. equi* dissemination to spleen occurred in 10/10 control mice but only 6/10 immunized mice (*χ*^2^ = 5.0; *p* = 0.0127). Immunized mice had an average of 100-fold less *S. equi* load in the spleen than control mice (Mann Whitney, *p* < 0.0001) (Fig. 8). *S. equi* numbers in the thigh were unaffected by CEP vaccination (Controls: median 7 × 10^7^, range (4.2–9.2) × 10^7^, Immunized: median 6.3 × 10^7^, range (2.6–9.2) × 10^7^). Serum IL-6 did not reflect the reduced bacterial load and remained similar between the two groups (Controls: median 515.7 pg/ml, range 177.6–1794 pg/ml, Immunized: median 552.3 pg/ml, range 153.6–1528 pg/ml).

## 4 Discussion

SpyCEP is the *S. pyogenes* cell envelope proteinase that is able to cleave and inactivate IL-8 and other CXC chemokines [11,15]. The surface location of SpyCEP and the high degree of conservation between isolates [15,16] makes it an ideal vaccine target. Indeed a whole genome proteomic and bioinformatic study identified SpyCEP (Spy0416) as a potential protective target promoting survival from an intranasal M23 infection, second only to M protein [10]. Studies in mice have demonstrated that SpyCEP plays an important role in disease [13,17] suggesting that neutralization of SpyCEP would be detrimental to the pathogen, although other studies could not confirm this [15,18]. In these preliminary studies we have demonstrated that immunization against SpyCEP induces neutralizing immunity and appears to prevent dissemination of disease from the site of infection. This was true for infection with *S. pyogenes* and *S. equi*.

Despite a global need, there is currently no licensed vaccine for GAS. The highly abundant surface M protein has been the most promising target but there have been problems associated with it. There are over 120 different M-types based on the N-terminal hypervariable region; antibodies directed to this region are opsonising but type specific [19]. Other regions of M protein generate antibodies that either are not opsonising or demonstrate cross reactivity with host tissue that may result in post streptococcal immune sequelae. SpyCEP does not demonstrate any sequence based serotype specificity and, although absolute levels of expression may vary, all clinical strains tested demonstrated SpyCEP activity that could be inhibited by antiCEP antibodies [16]. It seems likely that SpyCEP would be immunogenic in humans, since neutralizing antibodies to the protease are generated by natural infection, being found in pooled human intravenous immunoglobulin [Turner et al., unpublished].

We expressed and purified a 552aa portion of the SpyCEP protein spanning residues 35–587aa which begins immediately after the signal peptide and includes two of three catalytic domains. This recombinant protein (CEP) was administered intramuscularly, emulsified in adjuvant, and produced high systemic SpyCEP immunity. Immunization with CEP resulted in reduced dissemination of disease both from an intramuscular soft tissue infection and an intranasal upper respiratory infection. Clearance from the initial site of infection was not affected by vaccination although the study was designed to assess the effect on invasive disease and therefore the infection course was necessarily short. Full evaluation of CEP as a vaccine for *S. pyogenes* pharyngitis and carriage will require testing in an appropriate model of pharyngeal/tonsillar carriage.

*S. equi* and *S. zooepidemicus* both encode SpyCEP homologs that in this work have been shown to be functional homologs. *S. zooepidemicus* (*S. equi* subsp. *zooepidemicus*) is a mucosal opportunistic pathogen of a number of animals. Rarely it can also cause severe infections in humans such as bacteremia, meningitis and nephritis [20,21]. Despite carrying the gene for SzoCEP, protein expression and human IL-8 degrading activity was not detected in this strain. Cleavage of equine IL-8 was, however, detected suggesting that expression of SzoCEP is low in this strain. In contrast, SeCEP produced by *S. equi* was highly expressed throughout exponential growth both at the cell surface and released into the culture supernatant in a similar manner to SpyCEP produced by *S. pyogenes*. SeCEP cleaved both human IL-8 and equine IL-8 and cleavage of human IL-8 was inhibited by antiCEP serum confirming SeCEP specific proteolysis. The comparable expression pattern and function of SeCEP to SpyCEP suggested that the role of SeCEP in infection may be similar to SpyCEP, although the disease Strangles is more suppurative than that seen in clinical *S. pyogenes* disease. Immunization of mice with CEP reduced disease dissemination of *S. equi* from an intramuscular soft tissue infection. Although it did not completely inhibit infection dissemination, the disease produced by *S. equi* was more severe in mice than that caused by *S. pyogenes*. Strangles is predominantly a disease of the equine upper respiratory tract and lymph node system hence a full evaluation of CEP as a vaccine is required in an equine setting using intranasal administration.

## Figures and Tables

**Table 1:** Bacterial CFU from lung, nose and NALT tissue following intranasal infection with *S. pyogenes*.

	Controls		Immunized	
	Infected	CFU^a^	Infected	CFU
Lung	7/8	1.0 × 10^7^ (2.0 × 10^1^ to 1.9 × 10^7^)	7/8	1.4 × 10^6^ (0.2 × 10^1^ to 1.5 × 10^7^)
Nose	8/8	3.3 × 10^6^ (1.0 × 10^5^ to 9.5 × 10^6^)	8/8	4.2 × 10^6^ (6.0 × 10^5^ to 9.8 × 10^6^)
NALT	8/8	1.0 × 10^9^ (5.8 × 10^6^ to 1.7 × 10^10^)	7/8	3.0 × 10^9^ (5.0 × 10^6^ to 2.2 × 10^10^)

a Lung and nose: CFU/10 mg, NALT: CFU/NALT.
